# Supplementary material for: Osteogenic Commitment of Human Periodontal Ligament Cells Is Predetermined by Methylation, Chromatin Accessibility and Expression of Key Transcription Factors
Source: Cells. 2022 Mar 26;11(7):1126. doi: 10.3390/cells11071126 (PMC8997528; doi:10.3390/cells11071126)
Supplement: Supplementary file 1 [file cells-11-01126-s001.zip › cells-1635986-supplementary.pdf]

Supplementary Table S1. Primer sequences used for RT-qPCR.

Gene		Primer sequence 5'-3'	Reactions Conditions
ACTB	Forward<br>Reverse	CCAACCGCGAGAAGATGA<br>CCAGAGGCGTACAGGGATAG	95 ºC – 10"<br>61 ºC - 10"<br>72 ºC – 10"
OCT4	Forward<br>Reverse	CGCAAGCCCTCATTCAC<br>CATCACCTCCACCACCTG	95 ºC– 10"<br>60 ºC– 06"<br>72 ºC– 06"
PPARG	Forward<br>Reverse	GCTTATCTATGACAGATGTGATCT<br>AGCTTCTCCTTCTCGGC	95 ºC– 10"<br>55 ºC– 10"<br>72 ºC– 10"
SIX3	Forward<br>Reverse	CCTCCTCCTCTTCCTCCTCT<br>AGTGGTAGATGGTGGTTGGG	95 ºC – 10"<br>60 ºC - 20"<br>72 ºC – 10"
SP7	Forward<br>Reverse	ATGAGTGGGAAAAGGGAGGG<br>TCAACAACTCTGGGCAAAGC	95 ºC – 10"<br>60 ºC - 10"<br>72 ºC – 20"

Supplementary Table S2. Top 60 most differentially expressed genes at basal level between l- and h-PDLCs.

Symbol	Log2FC_High	Log2FC_Low
C1QL3	7.625	-6.019
CX3CR1	5.019	-5.220
DLX4	9.064	-7.405
EGFLAM	8.784	-5.718
FABP7	5.019	-5.746
FOXO6	6.893	-5.496
GRM3	5.996	-5.535
KRT18	7.231	-5.661
KRT81	7.441	-5.304
LRRC18	7.154	-5.384
PCDH1	5.535	-7.860
PLD4	6.688	-5.081
PLEKHN1	7.566	-5.788
PLVAP	6.450	-5.336
PSMB9	5.996	-7.824
SP7	8.370	-6.736
THNSL2	8.386	-9.274
TNNC1	5.306	-5.222
TSPAN18	6.010	-6.372
ABLIM2	-5.641	5.194
ACTN3	-7.207	5.628
ADH1C	-5.055	6.301
ADORA1	-8.120	6.640
BCHE	-5.931	8.511
BEX1	-6.627	5.087
BMP5	-6.847	5.351
CASQ1	-7.207	5.663
CDH18	-8.811	6.780
CHGB	-7.207	5.556
DDX25	-5.070	5.158
DNAJB7	-5.070	5.193
EPHA6	-6.847	5.058
ERICH5	-7.207	6.679
FIBCD1	-8.616	6.901
GALNT13	-12.680	11.179
GRID2	-6.847	5.191
IFNE	-6.627	5.085
IGFALS	-7.358	5.769
IGSF3	-7.558	5.722
IL1RN	-6.847	5.519
IL4	-6.847	6.034
IRX6	-7.940	6.395
JPH1	-7.125	5.697
L1TD1	-6.627	5.372
LRRTM1	-5.070	5.511
MAB21L2	-6.937	7.049
NKD1	-5.070	7.181
OCM	-7.207	5.886
OR5K2	-6.627	5.165
PCDH10	-5.899	5.883
PSG1	-7.734	5.329
PSMA8	-6.627	5.189
RSPH1	-5.641	5.084
SCN7A	-7.678	6.244
SIX3	-9.369	5.630
SLITRK5	-5.140	5.146
SMOC1	-6.733	6.101
TMEM196	-6.627	5.144
TMEM74	-6.847	5.340
TP53TG3D	-7.358	5.367

Symbol: gene identification
Log2FC_High: log2 fold change in h-PDLCs
Log2FC_Low: log2 fold change in l-PDLCs
Negative Log2FC: gene downregulated
Positive Log2FC: gene upregulated

Supplementary Table S3. Top 150 most differentially expressed genes at induced levels in l- and h-PDLCs

Symbol	Log2FC Low	Log2FC High
BST2	7.196	5.918
CHKB-CPT1B	6.436	5.774
FGL1	4.698	2.577
ART3	8.085	4.343
COMTD1	7.158	2.758
CRABP1	4.860	4.305
FCMR	4.653	4.074
GPM6B	4.503	3.449
CEMIP	4.321	3.487
FKBP5	5.762	3.715
ANO3	4.541	2.968
FCHO1	5.449	5.612
DGKB	4.289	6.676
FBXO43	3.639	6.930
ADGRF2	4.348	6.116
DPEP1	3.946	5.837
CXCL8	3.315	6.063
INMT-MINDY4	2.813	6.040
DKK1	2.550	6.567
COMP	3.819	5.213
DAPP1	3.542	5.073
CFAP65	2.980	5.238
BTLA	4.621	0.199
AURKA	4.582	0.199
ADRA1A	4.691	0.199
IGHV3-74	4.873	0.169
HLA-A	4.822	-0.156
C3	4.570	-0.092
CHI3L1	4.406	0.199
FPR2	4.397	0.199
FPR1	4.445	0.200
GNGT1	4.378	0.199
ATP13A4	4.353	0.250
IFITM5	4.502	0.199
ADAMDEC1	4.455	0.199
ALOX15B	5.365	0.199
AL121758.1	5.245	0.199
FMO3	5.119	-0.275
HLA-B	5.194	-1.021
ARHGDIG	4.974	-0.990
GLRA3	5.131	-0.804
CELA3B	5.134	-1.310
KCNN3	4.718	-1.161
GPR17	4.688	-0.785
CARNS1	4.400	-0.656
COMMD3-BMI1	5.942	0.199
BIN2	5.888	0.199
ADH4	6.249	0.199
DMKN	6.238	-0.170
CASC1	6.114	-0.425
KCNT1	5.617	-0.751
CDH5	4.438	1.773
HKDC1	4.367	1.499
HPD	4.822	0.974
FGR	4.419	1.145
ABCA2	5.364	0.584
ANKDD1A	5.164	0.753
CCDC13	5.945	0.965
GRIA2	6.679	0.707
C17ORF100	6.654	0.346
ANO2	7.906	0.290
ABL2	9.535	1.259
ERVMER34-1	6.593	-1.408
COL26A1	6.227	-1.335

Symbol	Log2FC Low	Log2FC High
AC069368.1	6.340	-2.353
CD101	8.129	-1.489
ASB2	4.445	-2.998
ATP6AP1	4.401	-3.091
DLEC1	4.908	-4.146
CTAGE15	4.561	-4.268
AC004223.3	4.328	-4.160
HSD17B11	4.375	-4.287
CHRFAM7A	5.545	-3.811
AMDHD1	6.250	-4.564
GMNC	8.830	-4.738
ARHGAP20	8.498	-4.993
BORCS8-MEF2B	7.399	-4.885
ANKRD26	9.161	-7.281
GNL1	8.281	-7.396
AC012184.2	7.842	-7.396
CORO7-PAM16	6.791	-6.630
AC090527.2	6.118	-5.955
AL022318.4	5.650	-6.168
ITIH5	5.371	-7.351
FAM183A	-0.368	5.432
GAL3ST3	-0.368	5.391
AC068946.1	-0.368	5.474
GNLY	-0.368	5.256
BOP1	-0.368	5.226
CYTH4	-0.368	5.068
FAM166B	-0.368	5.029
CLDN34	-0.368	4.952
CTXN3	-0.860	5.095
FIBCD1	-0.982	5.131
FOXD4L1	-0.697	5.036
FAM189A2	-0.677	5.387
KIF18B	-0.771	5.406
HYDIN	-1.106	5.829
CENPA	-1.052	5.669
BLOC1S5-TXNDC5	-0.711	5.980
AF241726.2	-1.364	5.210
CHRM3	-0.427	5.887
JSRP1	-0.368	5.811
AGAP2	-0.247	5.683
ALDOB	-0.417	5.583
CHGB	-0.042	6.048
AC008758.4	-0.567	6.896
ELMO3	-0.368	6.649
CRYM	1.656	5.119
GPR82	1.597	5.131
ADGRV1	1.565	5.501
IGKV2-30	2.087	5.809
AC104109.3	2.057	5.267
C1ORF87	2.374	4.869
APOBEC3B	0.697	5.478
DUXA	0.551	5.471
ASF1B	0.508	5.667
BLID	0.566	6.014
CDC45	1.091	5.678
BMP7	0.059	5.225
GYS2	0.311	4.899
HNRNPA1L2	-0.700	8.914
KIAA1107	-0.956	9.016
CNNM1	-0.368	8.265
CYP19A1	2.071	7.976
CDC25C	1.035	6.983

Symbol	Log2FC Low	Log2FC High
EPHB6	0.174	11.538
GOLGA8A	-2.813	12.699
HNRNPAB	5.902	8.637
FAM117B	6.236	8.179
CEMP1	4.420	9.641
CXCR5	4.826	12.415
EXPH5	-2.612	5.034
FUT6	-2.601	4.897
AL049844.3	-2.823	5.179
HLA-DOA	-2.826	4.986
CATSPERG	-2.219	5.532
DRGX	-2.687	5.468
FOXE3	-3.786	5.844
CRLF2	-3.797	5.754
DND1	-3.791	5.254
C1QTNF3-AMACR	-1.725	6.259
GPAT2	-2.709	6.267
CELF2	-4.461	5.599
AC104662.2	-4.501	5.009
C19ORF67	-5.132	4.898
CKAP2L	-5.383	6.646
GCH1	-6.244	6.206
KIF24	-8.416	8.937
FAM213B	-9.038	6.174

Symbol: gene identification
Log2FC_High: log2 fold change in h-PDLCs
Log2FC_Low: log2 fold change in l-PDLCs
Negative Log2FC: gene downregulated
Positive Log2FC: gene upregulated

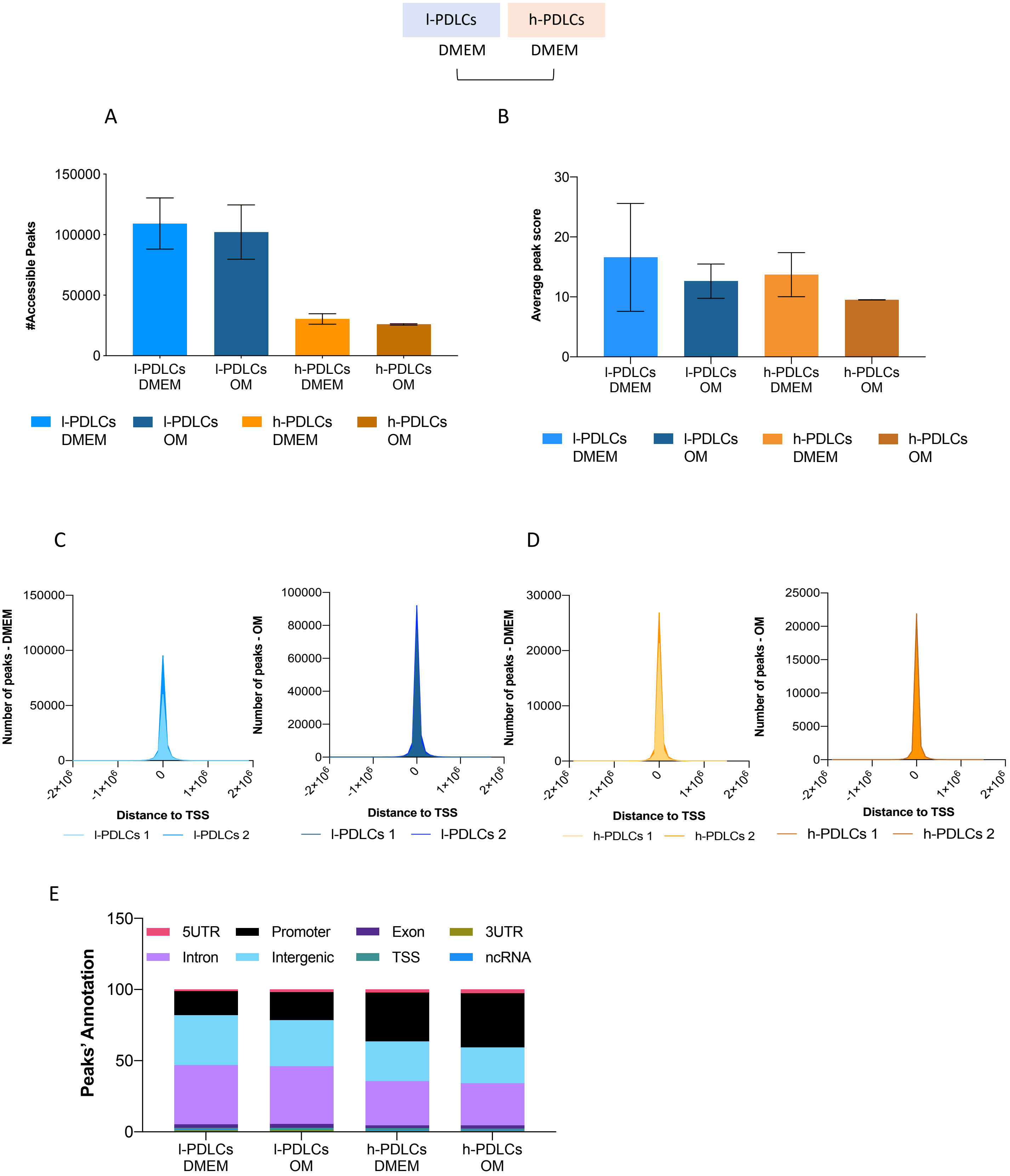

Supplementary figure S1. Chromatin accessibility in PDLCs. (A) Number of accessible chromatin peaks in l-PDLCs and h-PDLCs. (B) Average peak score at basal levels for l-PDLCs and h-PDLCs. (C, D) Distribution of peaks in relation to the nearest transcription start site (TSS) for each ATAC-seq replicate and experimental condition in l-PDLCs (C) and h-PDLCs (D). (E) Peaks' annotations shown as percentage of all peaks and compared between the experimental conditions. l-PDLCs, periodontal ligament cells showing low osteogenic potential *in vitro*; h-PDLCs, periodontal ligament cells showing high osteogenic potential *in vitro*.

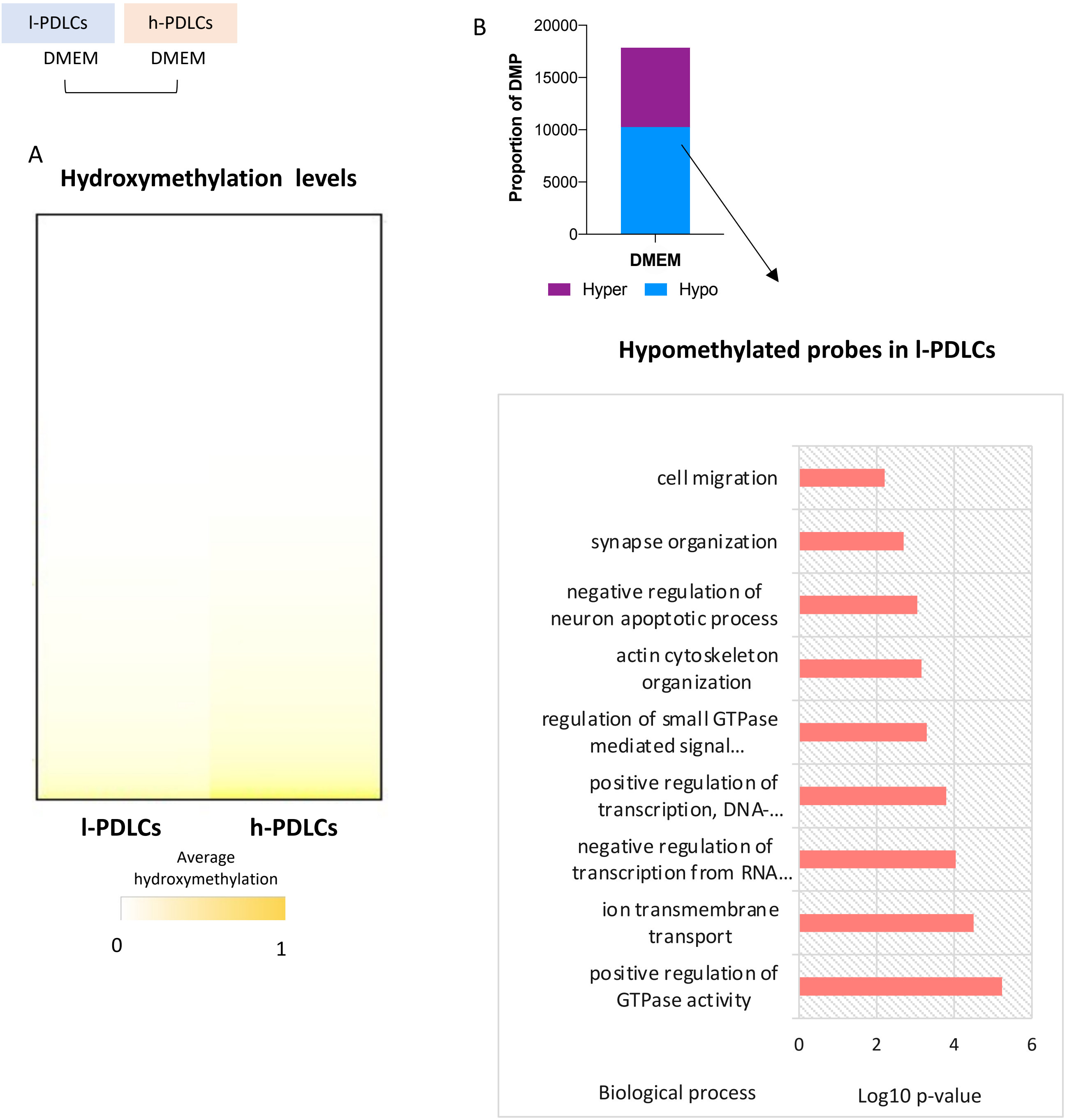

Supplementary figure S2. DNA hydroxymethylation of PDLCs at basal levels. (A) Heatmap showing DNA hydroxymethylation levels of all DNA methylation sites. (B) The proportion of 14,194 differentially methylated probes at day 10 of osteogenic stimulations *in vitro* in l-PDLCs (upper graph) and biological processes related to this subset. l-PDLCs, periodontal ligament cells showing low osteogenic potential *in vitro*; h-PDLCs, periodontal ligament cells showing high osteogenic potential *in vitro*.

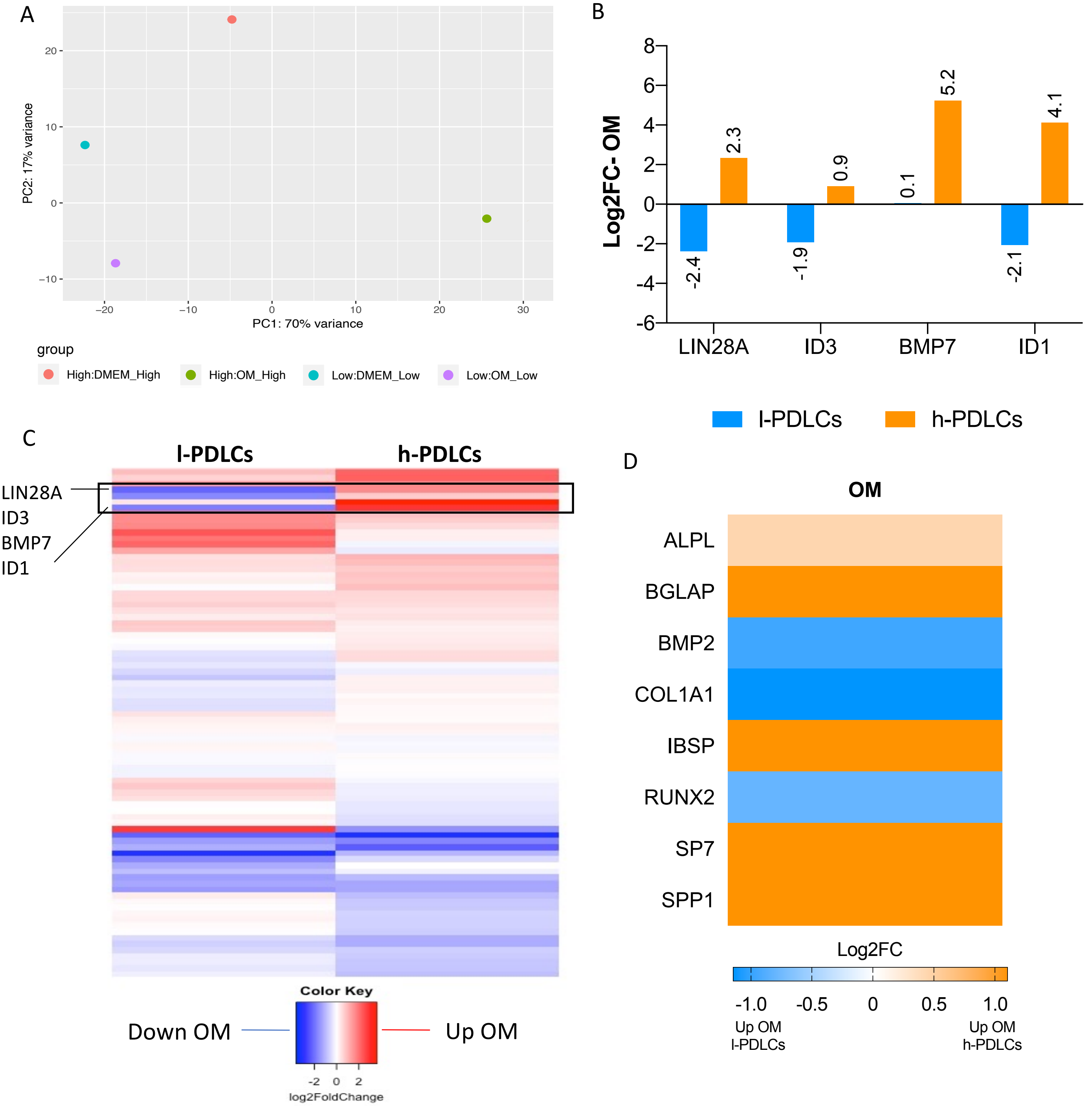

Supplementary figure S3. Transcriptional analysis of osteogenic markers. (A) PCA plot of RNA-seq data showing the four experimental conditions: h-PDLCs in DMEM, h-PDLCs in OM, l-PDLCs in DMEM and l-PDLCs in OM. For each condition, three independent replicates were first performed and then pooled for sequencing. The variability represented by the first component clearly separates the h-PDLCs after OM stimulation from the other samples. (B) OM-induced gene expression changes in RNA-seq data in l-PDLCs and h-PDLCs. The panel represents genes responsible for controlling osteoblast lineage progression as described in *"Genetic and transcriptional control of bone formation"* by Javed et al. (2010). (C) Gene expression changes for four genes most differentially up-regulated by OM in h-PDLCs (as highlighted in (B)). (D) Comparison of gene expression levels of classical osteogenic markers between l- and h-PDLCs at day 10 of osteogenic stimulation. l-PDLCs, periodontal ligament cells showing low osteogenic potential *in vitro*; h-PDLCs, periodontal ligament cells showing high osteogenic potential *in vitro*.

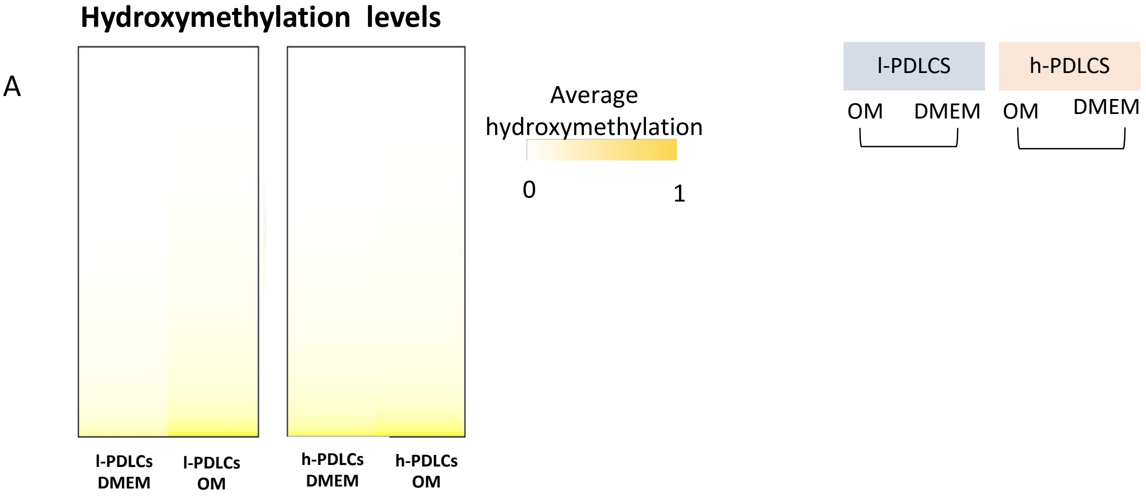

B

OM			
Probe	l-PDLCs DeltaBeta	Gene	Role
cg09391665	0.594	SEC23IP	The encoded protein is localized to endoplasmic reticulum exit sites and plays a critical role in ER-Golgi transport as part of the multimeric coat protein II complex
cg11382054	0.457	PPFIBP1	May regulate the disassembly of focal adhesions.
cg03771702	0.425	HM13	Catalyzes intramembrane proteolysis of some signal peptides after they have been cleaved from a preprotein, resulting in the release of the fragment from the ER membrane into the cytoplasm.
cg01832737	0.416	PDE10A	Plays a role in signal transduction by regulating the intracellular concentration of cyclic nucleotides
cg05960822	0.391	EBF1	For instance, positively regulates many B-cell specific genes including BCR or CD40 while repressing genes that direct cells into alternative lineages, including GATA3 and TCF7 for the T-cell lineage. In addition to its role during lymphopoiesis, controls the thermogenic gene program in adipocytes during development and in response to environmental cold

C

OM			
Probe	h-PDLCs Delta Beta	Gene	Role
cg14683065	0.46622582	LRRK1	The encoded protein is thought to play a role in the regulation of bone mass. Mice lacking a similar gene showed severe osteopetrosis, increased bone mineralization and decreased bone resorption.
cg04049542	0.35763966	C11orf80	Together with SPO11, mediates DNA cleavage that forms the double-strand breaks (DSB) that initiate meiotic recombination. The complex promotes relaxation of negative and positive supercoiled DNA and DNA decatenation through cleavage and ligation cycles.
cg01997461	0.35682086	FARP1	Functions as guanine nucleotide exchange factor for RAC1. Plays a role in the assembly and disassembly of dendritic filopodia, the formation of dendritic spines, regulation of dendrite length and ultimately the formation of synapses.
cg14313797	0.34804238	CAMK1D	This gene is a member of the calcium/calmodulin-dependent protein kinase 1 family, a subfamily of the serine/threonine kinases. In vitro, phosphorylates transcription factor CREM isoform Beta.
cg10582795	0.34776656	RBMS1	These proteins have been implicated in such diverse functions as DNA replication, gene transcription, cell cycle progression and apoptosis. Probably has a role in DNA replication.

Supplementary figure S4. DNA hydroxymethylation in PDLCs following osteogenic induction. (A) Heatmap showing DNA hydroxymethylation levels of 850,000 DNA methylation sites at basal state and after 10 days of osteogenic induction. (B) Top 5 most DNA hydroxymethylated probes in l-PDLCs after osteogenic induction. (C) Top 5 most hydroxymethylated probes in h-PDLCs at induced levels. l-PDLCs, periodontal ligament cells showing low osteogenic potential *in vitro*; h-PDLCs, periodontal ligament cells showing high osteogenic potential *in vitro*. DMEM, standard culture medium; OM, osteogenic medium.
